# What Are the Proteolytic Enzymes of Honey and What They Do Tell Us? A Fingerprint Analysis by 2-D Zymography of Unifloral Honeys

**DOI:** 10.1371/journal.pone.0049164

**Published:** 2012-11-07

**Authors:** Rocco Rossano, Marilena Larocca, Teresa Polito, Anna Maria Perna, Maria Carmela Padula, Giuseppe Martelli, Paolo Riccio

**Affiliations:** 1 Department of Biology, Defence and Agro-Forestal Biotechnology and Centre of Bioproteomics, University of Basilicata, Potenza, Italy; 2 Department of Animal Production Sciences, University of Basilicata, Potenza, Italy; Aligarh Muslim University, India

## Abstract

Honey is a sweet and healthy food produced by honeybees (*Apis mellifera* L.) from flower nectars. Using bidimensional zymography, we have detected the, until now unrevealed, proteolytic activities present in row honey samples. The resulting zymograms were specific for each type of the four unifloral honey under study, and enzymes were identified as serine proteases by the use of specific inhibitors. Further, using bidimensional electrophoresis, we have shown that honey proteases are able to degrade the major Royal Jelly proteins and in particular MRPJ-1, the protein that promotes queen differentiation in honeybees. Our findings open new perspectives for the better understanding of honeybee development, social behaviour and role in honey production. The now discovered honey proteases may influence honey properties and quality, and bidimensional zymograms might be useful to distinguish between different honey types, establish their age and floral origin, and allow honey certification.

## Introduction

Honey is the natural product processed by honeybees (*Apis mellifera* L.) from flower nectars or plant secretions, by aid of their own secretions. Honey is a nutritious, sweet and healthy food, composed mostly of the sugars glucose and fructose. Minor components of honey include proteins (0.25–0.5%), organic acids, aminoacids, vitamins and flavonoids. The chemical composition of honey - either from single or multi-floral species – depends mainly on beekeeper ability, on the botanical and geographical origin of the nectars, and on the different climatic and environmental conditions. Several natural components of honey have been used as a marker to determine floral, geographical and botanical origin of the product: phenolic compounds [1], [2], proteins [3], aminoacids [4], oligoelements [5] and carbohydrates [6].

Honey proteins can originate from nectar and pollen of flowers, or occasionally from the sap of plants, but more often they derive from secretions of cephalic glands of honeybees. Most important are the nine Major Royal Jelly proteins (MRJP). Among them, MRJP1 is likely to promote liver regeneration and to have a cytoprotective action on hepatocytes [7], MRJP3 can exhibit potent immunoregulatory effects *in vitro* and *in vivo* [8], and both MRJP4 and MRJP5 are important sources of essential aminoacids [9].

Enzymes involved in carbohydrate metabolism or in defence are well known: diastase (amylase), invertase (saccharase), glucosidase, glucose oxidase and catalase [10], [11]. With regard the proteolytic enzymes of honey, it is known that honeybees use three midgut endopeptidases (trypsin, chymotrypsin and elastase) and the exopeptidase leucineaminopeptidase to digest dietary proteins [12], but the presence of proteolytic enzymes in honey has not been described yet. Therefore, the objective of our study was to investigate about the presence of proteolytic activities in honey and to evaluate their effects on honey protein profile. This is an important aspect when proteins are used as chemical markers of the geographical and floral origins of honeys [13]–[17].

In this study, we have analysed four commercial unifloral honeys: Orange (*Citrus* sp), Chestnut (*Castanea sativa* Miller.), Eucalyptus (*Eucalyptus* sp) and Italian Sainfoin (*Hedysarium coronarium* L.), commonly known in Italy as “Sulla”. Honeys were manufactured in two different geographical areas of the Region Basilicata (Southern Italy). For each honey type, four samples were analyzed: two from the Province of Matera (MT) (honey_MT1_ and honey_MT2_) and two from the Province of Potenza (PZ) (honey_PZ1_ and honey_PZ2_). In total, 16 samples were analysed in duplicate. For the analysis, we have applied bidimensional zymography (2-DZ), a technique that allows the detection of the entire proteolytic network present in a biological sample, including isoforms and post-translational variants.

In 2-DZ, proteases are separated by isoelectric focusing (IEF), according to their pIs in the 1^st^ dimension, and by SDS gel electrophoresis, according to their Mw, in the 2^nd^ dimension. The polyacrylamide gel for SDS electrophoresis is copolymerized with an appropriate protein substrate (here gelatin), which is degraded by the enzymes once reactivated after the run. Proteolytic enzymes, their isoforms and post-translational variants, present in the extracts are detected as clear, unstained and translucent spots on a dark or blue background. Therefore, 2-DZ represents a useful technique to detect the whole pattern of proteolytic enzymes present in the sample under study [18]–[20].This study describes for the first time the proteolytic enzymes present in honey and their effects on honey proteins.

## Results and Discussion

### Protein Content and Proteolytic Activity in Different Honeys

Table 1 shows protein content and total proteolytic activity of sixteen commercial unifloral honey samples of four different floral origins and from two different geographical areas, taken for the study. The highest protein content was found in *eucalyptus* honey, whereas the highest values of total proteolytic activity were detected in the extracts obtained from *chestnut* honey. No differences were found between the proteolytic activities of *orange* and *sulla* honeys in their corresponding samples of the same botanical origin but manufactured in different areas. By contrast, *chestnut* honeys, from Potenza (honeys_PZ_) showed lower activities when compared with those from Matera (honeys_MT_), whereas the *eucalyptus* samples, from Potenza showed higher proteolytic activities when compared with those from Matera.

**Table 1:** Protein content and total proteolytic activity of honey samples of different floral origins.

Sample	Honey samples	Protein^a^	Total proteolytic activity^b^
		(mg prot g honey^−1^)	(mU mg prot^−1^)
1	Orange_MT1_ * (*Citrus*)	0.62*^c^*±0.07	14.47*^e^*±1.05
2	Orange_MT2_ (*Citrus*)	0.58*^c^*±0.08	14.80*^e^*±1.16
3	Orange_PZ1_ (*Citrus*)	0.66*^c^*±0.11	15.05*^e^*±1.31
4	Orange_PZ2_ (*Citrus*)	0.72*^d^*±0.10	15.23*^e^*±0.44
5	Eucalyptus_MT1_ (*Eucalyptus* sp)	0.91*^d^*±0.13	15.96*^e^*±0.95
6	Eucalyptus_MT2_ (*Eucalyptus* sp)	0.93 *^d^*±0.15	14.97 *^e^*±1.08
7	Eucalyptus_PZ1_ (*Eucalyptus* sp)	1.24*^d^*±0.14	21.36*^f^*±1.27
8	Eucalyptus_PZ2_ (*Eucalyptus* sp)	1.24*^d^*±0.14	19.06*^f^*±0.88
9	Sulla_MT1_ (*Hedysarium coronarium*)	0.70*^c^*±0.12	15.22*^e^*±0.48
10	Sulla_MT2_ (*Hedysarium coronarium*)	0.84*^c^*±0.22	16.40*^e^*±1.21
11	Sulla_PZ1_ (*Hedysarium coronarium*)	0.68*^c^*±0.11	15.53*^e^*±0.09
12	Sulla_PZ2_ (*Hedysarium coronarium*)	0.78*^c^*±0.91	16.03*^e^*±0.68
13	Chestnut_MT1_ (*Castanea sativa*)	0.71*^c^*±0.09	32.49*^g^*±1.21
14	Chestnut_MT2_ (*Castanea sativa*)	0.60*^c^*±0.06	36.43*^g^*±2.05
15	Chestnut_PZ1_ (*Castanea sativa*)	0.69*^c^*±0.16	25.29*^h^*±3.11
16	Chestnut_PZ2_ (*Castanea sativa*)	0.59*^c^*±0.10	27.61*^h^*±1.89

a Protein content was determined by the method of Bradford.

b Total proteolytic activity is expressed in mU mg prot^−1^, where mU corresponds to the amount of enzyme yielding 0.001 units of absorbance at 280 nm per min (pH 7.5, T = 30°C),

Proteolytic activity was assessed on gelatine in solution (mean ± SD, n = 6).

c,d,e,f,g,h The mean values with different letters superscript (in the same column) are significantly different (*p*<0.05) as analyzed by one way analysis of variance (ANOVA).

* The lower script code indicates the geographical origin of the honeys: MT (Province of Matera) and PZ (Province of Potenza).

### 2-D Zymography (2-DZ)

Non-reducing 2-D zymography was performed in duplicate to detect the proteolytic network present in the extracts obtained from the honey samples.

**Figure 1:**
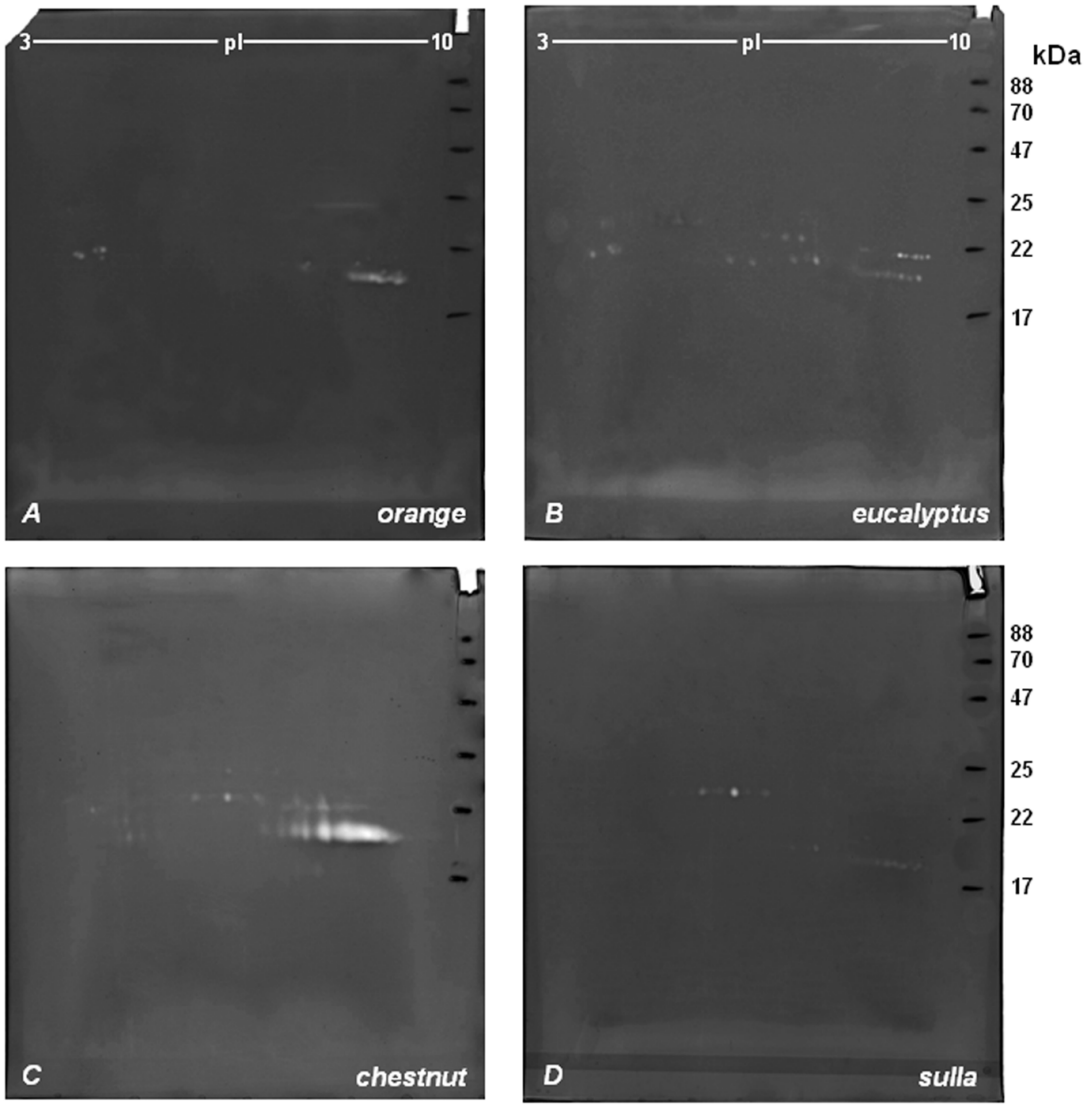
2-D zymography (2-DZ) of the proteolytic activities present in honey_MT1_ extracts. Proteolytic activities were detected by 2-D zymography on gels copolymerized with gelatin. Gels: A (*orange* honey), B (*eucalyptus* honey), C (*chestnut* honey) and D (*sulla* honey). 80 µg of proteins were applied to each gel.

Figure 1 show the representative 2-DZ from the four types of honeys_MT1_. As shown in Fig. 1., 2-D zymograms were different for each of the four honey varieties.

*Orange* honey extracts (Fig. 1, gel A) revealed the presence of 3 groups ofclear, unstained digestion spots. The 1^st^group was made of some unresolved spots of 24 kDa, at pI between 8.2 and 8.8; the 2^nd^ group was located in the acidic region of the gel at around 20 kDa, at pI between 4.2 and 4.4; finally the 3^rd^ group, characterized by the highest proteolytic activity, consisted of 9 spots of 19 kDa, at pI between 8.5 and 9.3.

*Eucalyptus* honey extracts (Fig. 1, gel B) showed 3 different groups of digestion spots, each consisting of a double row of spots. The 1^st^ group, consisting of 5 spots with molecular mass between 22.5 and 24 kDa and pI between 4.2 and 4.5; the 2^nd^ consisting of 10 spots between 21 and 23.6 kDa and pI between 6.4 and 8; and finally the 3^rd^ group consisting of 12 spots between 19 and 21.5 kDa and pI between 8.5 and 9.4.

*Chestnut* honey extracts (Fig. 1, gel C) showed 4 groups of spots: the 1^st^ in the central area of the gel formed by 4 well-resolved spots of 22.8 kDa with pI between 5.9 and 6.6; the 2^nd^ consisting of 5 spots of 22 kDa with pI between 7.65 and 8.10; the 3^rd^ (more evident in gel A than in gel B), with molecular mass between 19 and 22 kDa and pI between 4.22 and 4.82; and finally the 4^th^ group, characterized by an intense proteolytic activity, with molecular mass of 20 kDa and pI between 7.11 and 9.10.

Finally, *Sulla* honey extracts showed the presence of 2 groups of spots: the 1^st^ of 24 kDa consisting of 6 well resolved isoelectric variants, with pI between 4.95 and 6.80; and the 2^nd^ appearing as a horizontal streaking of 19 kDa with pI between 8.9–9.6.

**Figure 2:**
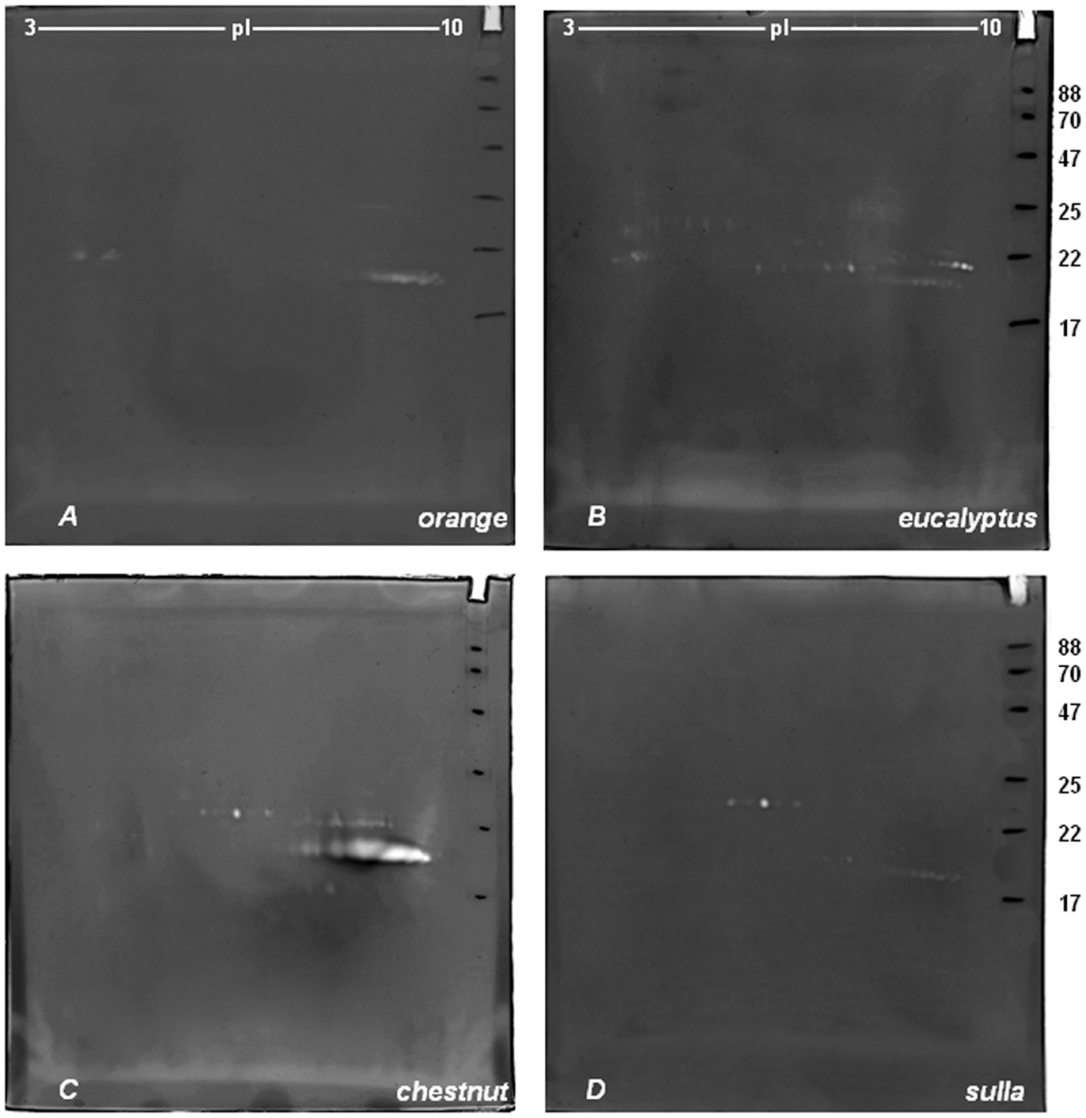
2-D zymography (2-DZ) of the proteolytic activities present in honey_MT2_ extracts. Proteolytic activities were detected by 2-D zymography on gels copolymerized with gelatin. Gels: A (*orange* honey), B (*eucalyptus* honey), C (*chestnut* honey) and D (*sulla* honey). 80 µg of proteins were applied to each gel.

In Fig. 2 are showed the representative 2-DZs of the four extracts obtained from the honeys_MT2_. All zymograms were very similar to those observed for the honey_MT1_ except for a few minor aspects. In particular, *Orange* honey extract (Fig. 2, gel A),in correspondence to the 3^rd^ group of spots (19 kDa and pI between 8.5 and 9.3) revealed, respect to honey_MT1_ (Fig. 1, gel A), the presence of two other spots at pI 9.4–9.5.

*Eucalyptus* honey extracts (Fig. 2, gel B) showed 3 different groups of digestion spots, each consisting of a double row of spots such as in Fig. 1. With respect to honey_MT1_, the 3 groups of spots showed the same zymographic profile except for the intensity of digestion spots detected at 21.5 kDa and pI between 8.5 and 9.4, that were higher than those observed in Fig. 1.

No differences were observed for *chestnut* (Fig. 2, gel C) and *sulla* (Fig. 2, gel D) honey_MT2_, when compared with the first set of samples from Matera.

**Figure 3:**
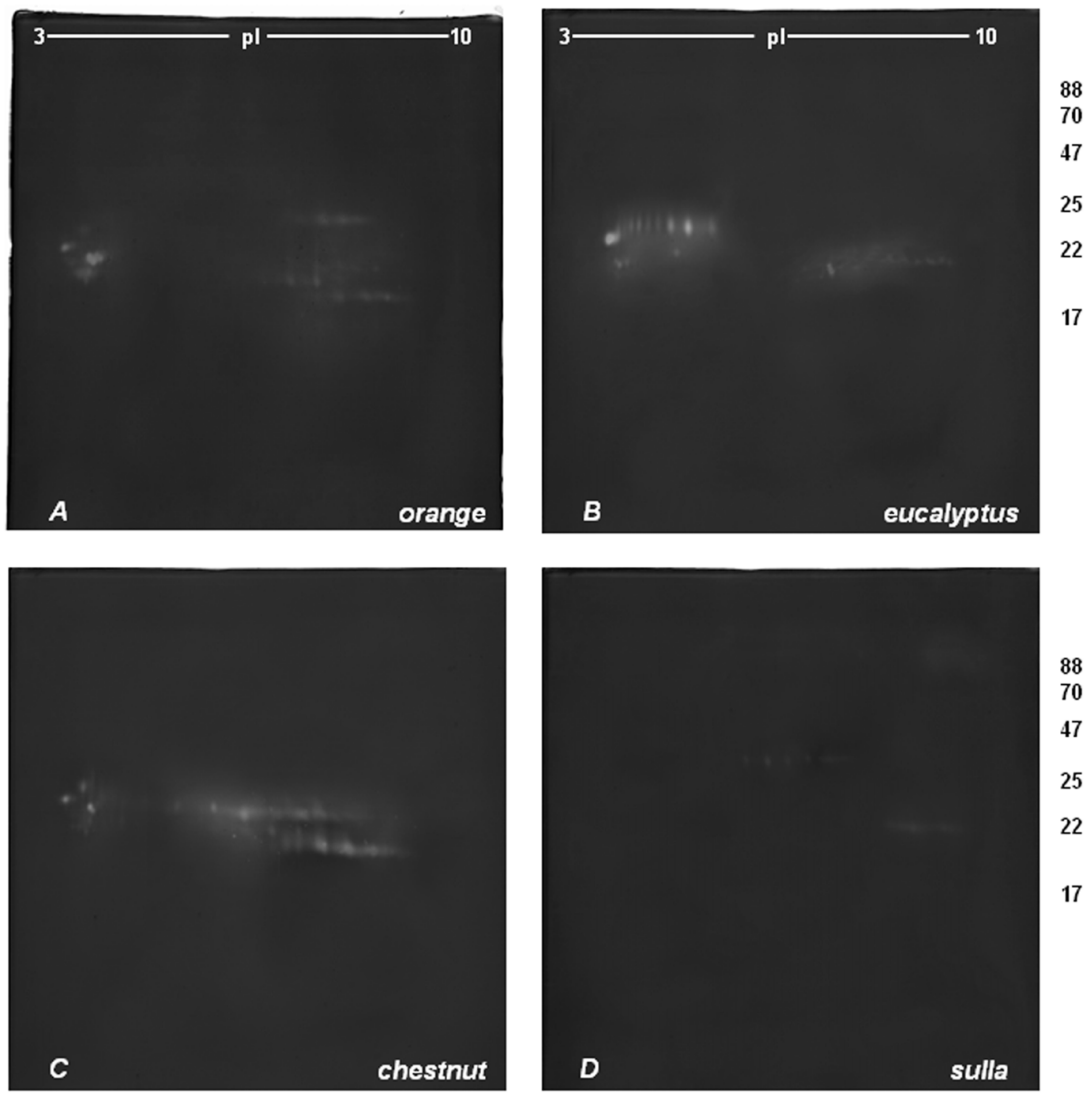
2-D zymography (2-DZ) of the proteolytic activities present in honey_PZ1_ extracts. Proteolytic activities were detected by 2-D zymography on gels copolymerized with gelatin. Gels: A (*orange* honey), B (*eucalyptus* honey), C (*chestnut* honey) and D (*sulla* honey). 80 µg of proteins were applied to each gel.

**Figure 4:**
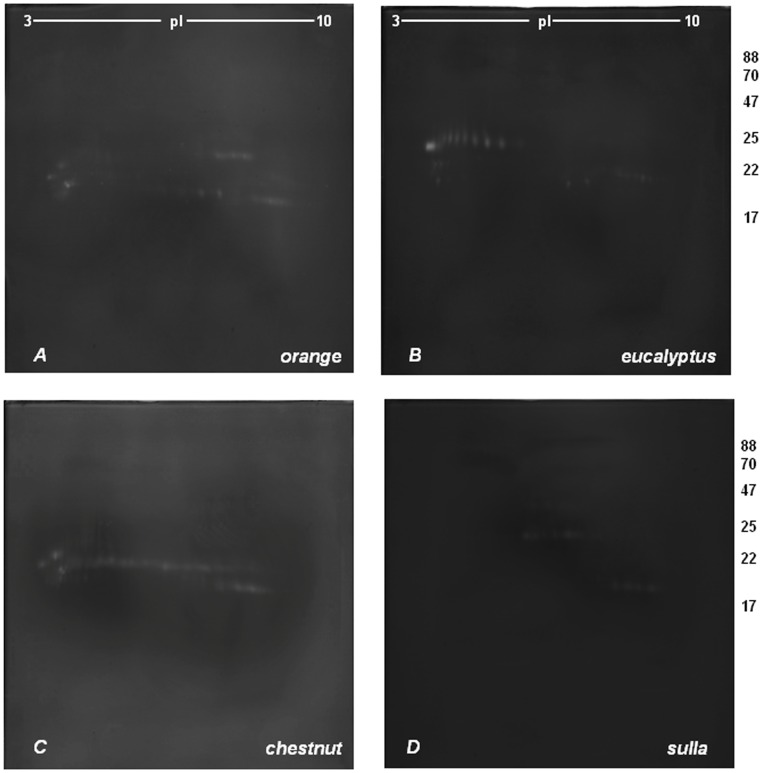
2-D zymography (2-DZ) of the proteolytic activities present in honey_PZ2_ extracts. Proteolytic activities were detected by 2-D zymography on gels copolymerized with gelatin. Gels: A (*orange* honey), B (*eucalyptus* honey), C (*chestnut* honey) and D (*sulla* honey). 80 µg of proteins were applied to each gel.

Figures 3 and 4 show the representative 2-DZs from the four types of honeys_PZ1_ and honeys_PZ2_. In both sets, the zymograms were different for each of the four honey varieties. No difference were found, between the honeys of the same botanical origin.

In *orange* honey extracts (Figs. 3–4, gels A) the same groups of digestion spots was present as in honeys_MT_, with the only difference that three additional spots were present at 20 kDa and pI of 7.9–8.25. Regarding the relative activity of proteolytic spots, in honeys_PZ_, spots located in the acidic region of the gel at around 20 kDa and at pI between 4.2 and 4.4 showed higher activity than those observed in honeys_MT_ (Figs. 1–2, gels A). No differences were observed in other spot groups.

The *Eucalyptus* honeys_PZ_ (Figs. 3–4, gels B) showed a different proteolytic pattern with respect with those from Matera (Figs. 1–2, gels B). They were characterized by the presence of digestion spots in the same three zones of the gels observed in the honeys_MT_ with some differences. In particular, in the acidic region corresponding to the molecular mass of 24 kDa were detected 7 additional distinct spots with pI between 4.5 and 5.5. No differences were observed in the2^nd^ region consisting of 10 spots between 21 and 23.6 kDa and pI between 6.4 and 8. Finally, in the basic region, only the spot row of 21.5 kDa was detected.

Regarding the *Chestnut* honey_PZ_ extracts (Figs. 3–4, gels C) no differences were detected respect to honeys_MT_ (Figs. 1–2, gels C), except for the 4^th^ group of spots, showing a lower proteolytic activity when compared to the corresponding extracts from Matera.

Finally, *Sulla* honey_PZ_ extracts (Figs. 3–4, gels D), showed zymographic profiles similar to those observed in honeys_MT_ (Figs. 1–2, gels D).

Our results indicate that the 2-DZ technique can allow to discriminate between honeys with different unifloral origin. The influence of geographical origin appears to be relevant in the case of *Eucalyptus* honeys, is less important in *orange* honeys, and is not detectable in the case of *sulla* and *chestnut.*

Class and type of proteases were determined by using specific inhibitors: PMSF, for serine proteinases; iodoacetamide, for cysteine proteinases, and EDTA, for metallo-proteinases.

**Figure 5:**
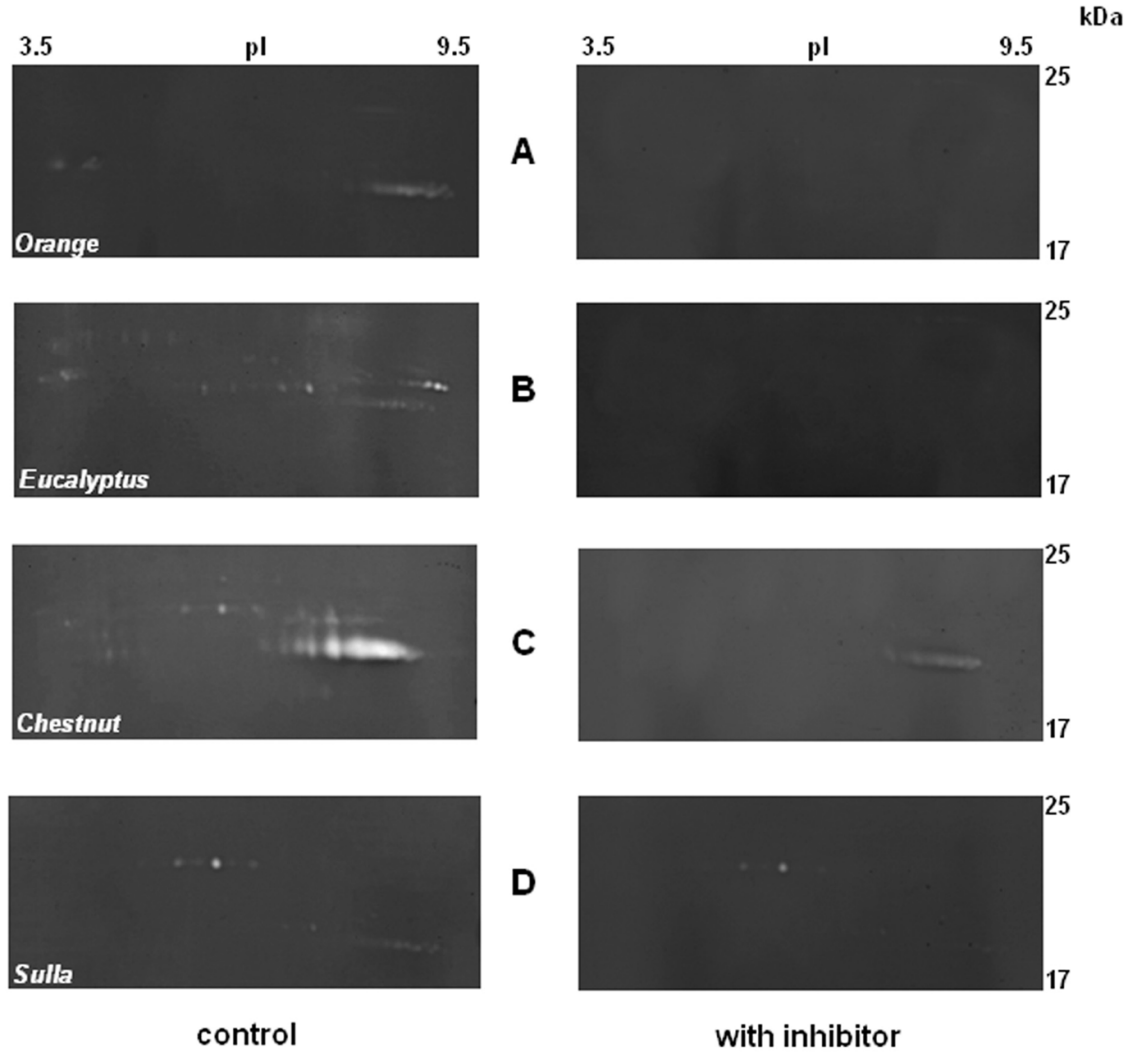
Specific inhibition by PMSF of the proteolytic activities present in honey_MT1_ extracts. 2-D zymography of the honey extracts, in the absence (control, left panels) and in the presence (right panels) of 1.5 mM PMSF, inhibitor of serine proteases. Gels: 1 (*orange*), 2 (*eucalyptus*), 3 (*chestnut*) and 4 (*sulla*).Only the regions of the gels with isoelectric point between pH 3.5–9.5 and molecular mass between 17–25 kDa are shown.

As shown in Fig. 5, PMSF inhibited almost completely the proteolytic activities of *Orange* and *Eucalyptus* honey extracts (panels A and B, on the right), and only partially those of the *Chestnut* and *Sulla* honey extracts (panels C and D, respectively). No inhibition was observed in the presence of EDTA or iodoacetamide (data not shown). Specific inhibitors of aspartic proteases were not tested, because gelatin does not represent a good substrate for aspartic proteases. Other inhibitors were tested only on unidimensional zymoghraphy (1-DZ): TLCK, trypsin specific, was as effective as PMSF.

E-64, specific for cysteine proteases; and 1,10 phenantroline, specific for metallo-proteinases, had no effect at the concentration used. All the inhibitors mentioned above, as well as Pepstatin A, were ineffective in gelatin solution assays.

On the basis of the results obtained, most of the proteolytic enzymes could be assigned to the class of serine proteases.

Honey proteases may originate from nectar, pollen or from the secretions of cephalic glands of honeybees. In honeybees, proteases - and in particular serine proteases - besides being involved in the digestive process, perform other important functions in development, immune response and defence against pathogens [21], [22]. In the genome of *Apis mellifera*, 46 genes encoding for serine proteases and 12 genes, *in silico* predicted as serine protease-like [21], have been identified. Within this protease family, trypsin and chymotrypsin are the most represented members.

Data regarding Mw and theoretical pIs, calculated by applying the “Compute pI/Mw” tool available at Expasy site (http://web.expasy.org/) on 58 protein sequences related to serine proteases of *Apis mellifera*, available in “RefSeq protein database” at NCBI (http://www.ncbi.nlm.nih.gov/protein), are very similar to those observed experimentally in the gel regions with pIs between 7.5 and 9.5. In particular, the various proteolytic activities found in the gels could correspond to different forms of trypsin: (XP-001123271.2: Trypsin-1, Mw: 21.92 - pI: 9.22; XP-003251667.1:Trypsin-3-like protein A, Mw: 25.60 - pI: 9.31; XP-001120923.1:Trypsin 3A1, Mw: 29.18 - pI: 9.32; XP-001120454.2: Trypsin-7, Mw: 18.46 - pI: 8.41) or chymotrypsin (XP-394370.1: Chymotrypsin-1, Mw: 28.06 - pI: 7.59; ACE75344.1:Chymotrypsin-like protein, Mw: 29.24 - pI: 8.96; XP-624680.3: Chymotrypsin-2, Mw: 27.66 - pI: 8.44). Spots observed in the acidic region might be proteases of vegetal origin, typical of floral varieties, resulting from the breakage of pollen grains, as the presence of proteolytic enzymes in the pollen grain was found as early as over 100 years ago [23]. However, the proteomic analysis of the honey extracts, did not reveal the presence of proteins of plant origin, probably because they were degraded by honeybee proteases and were present in undetectable amounts.

On the other hand, if the presence of proteolytic enzymes of vegetal sources is not relevant, and the honey bees are from the same species, what determines the diversity of the 2-DZ proteolytic patterns observed in the different unifloral honeys?

Pollens are collected by the honeybee to provide a source of protein, carbohydrate, vitamins and minerals for developing bee larvae. Honeybees secrete proteolytic enzymes into the midgut and digest the various proteins of pollen grains into peptides and aminoacids. Different pollen types have different kinds and amounts of proteins that could induce in a different way the honeybee expression of specific proteolytic enzymes, thus leading to the definition of the typical 2-DZ proteolytic maps observed in different unifloral honeys as well as to the different values of total proteolytic activity.

### Effect of the Proteolytic Enzymes on the Major Honey Proteins

**Figure 6:**
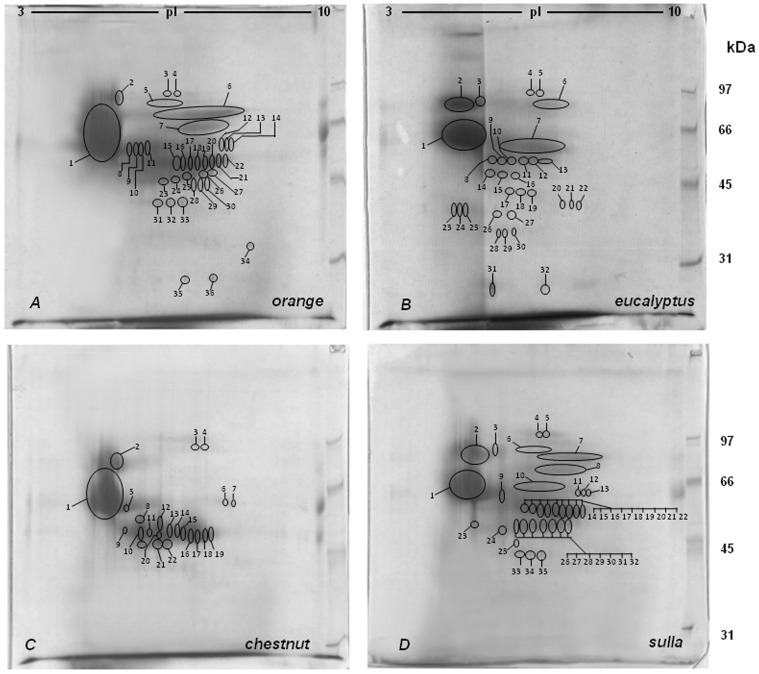
Bidimensional electrophoresis (2-DE) of proteins present in different unifloral honey_MT1_ samples. 2-DE analysis (IEF: linear pH gradient of 3–10; SDS-PAGE: 10% polyacrylamide). Coomassie stained gels: A (*orange*), B (*eucalyptus*), C (*chestnut*) and D (*sulla*). 300 µg of proteins were applied to each gel. Numbers corresponds to the protein spots identified by MALDI-ToF MS analysis.

The degradative action of proteolytic enzymes on the major proteins of honey was evaluated by bidimensional gel electrophoresis (2-DE). The protein profiles of each honey extract were analyzed in duplicate. Figure 6 shows the 2-DE analysis on 10% polyacrylamide gels of high-Mw proteins of honey_MT1_ extracts. All spots detected in the four honey types were excised from the gels, digested with trypsin and analyzed by MALDI-ToF MS. The protein pattern of all the honeys was complex, due both to the high abundance and heterogeneity of MRJPs, as well as to the presence of degradative events or post-translational modifications such as glycosylation [24], [25]. All proteins identified in the four honey types belonged to two groups of *Apis mellifera* proteome: the MRJP family and the enzymes involved in carbohydrate metabolism. The identified proteins were listed in Tables 2, 3, 4 and 5. In particular, 5 proteins belonged to the family of the major royal jelly proteins 1–5: *MRJP1*, AC XP_393380, Mr 49.31, pI 5.10; *MRJP2*, AC XP_393061, Mw 51.44, pI 6.83; *MRJP3*, AC XP_391893, Mw 61.62, pI 6.47; *MRJP4*, AC XP_001119880, Mw 52.88, pI 5.89; and AC XP_396121, Mw 70.19, pI 5.90. In all samples, MRJP1 was the dominant protein. Several variants were observed in the case of MRJP2, indicating different degrees of glycosylation and proteolysis. MRJP3 was detected in all samples as horizontal streaking, whereas the MRPJ4 and MRJP5 were identified in all samples as a single spot. Regarding to the enzymes involved in carbohydrate metabolism, two proteins were identified: glucose oxidase (AC XP_392386, Mw 70.19, pI 5.90) and alpha-glucosidase (AC XP_392790, Mw 65.69, pI 5.06).

**Table 2:** Protein identification in the honey orange_MT1_ extract^a^.

Spot	Protein Name	Accession	Peptides	Cov. (%)	Score^b^	pI	kDa
1	major royal jelly protein 1 [Apis mellifera]	XP_393380	10	23	91	5.10	49.31
2	alpha-glucosidase precursor [Apis mellifera]	XP_392790	7	19	74	5.06	65.69
3	Glucose oxidase [Apis mellifera]	XP_392386	11	22	107	6.48	67.90
4	Glucose oxidase [Apis mellifera]	XP_392386	8	19	69	6.48	67.90
5	major royal jelly protein 5[Apis mellifera]	XP_396121	16	25	121	5.90	70.19
6	major royal jelly protein 3 [Apis mellifera]	XP_391893	10	25	100	6.47	61.62
7	major royal jelly protein 3 [Apis mellifera]	XP_391893	11	27	108	6.47	61.62
8	major royal jelly protein 4 [Apis mellifera]	XP_001119880	9	20	87	5.89	52.88
9	major royal jelly protein 2 [Apis mellifera]	XP_393061	7	18	61	6.83	51.44
10	major royal jelly protein 2 [Apis mellifera]	XP_393061	9	21	67	6.83	51.44
11	major royal jelly protein 2 [Apis mellifera]	XP_393061	9	21	67	6.83	51.44
12	major royal jelly protein 3 [Apis mellifera]	XP_391893	14	31	172	6.47	61.62
13	major royal jelly protein 3 [Apis mellifera]	XP_391893	11	27	108	6.47	61.62
14	major royal jelly protein 3 [Apis mellifera]	XP_391893	8	21	71	6.47	61.62
15	major royal jelly protein 2 [Apis mellifera]	XP_393061	7	18	68	6.83	51.44
16	major royal jelly protein 2 [Apis mellifera]	XP_393061	12	33	106	6.83	51.44
17	major royal jelly protein 2 [Apis mellifera]	XP_393061	9	21	88	6.83	51.44
18	major royal jelly protein 2 [Apis mellifera]	XP_393061	8	19	66	6.83	51.44
19	major royal jelly protein 2 [Apis mellifera]	XP_393061	5	12	58	6.83	51.44
20	major royal jelly protein 2 [Apis mellifera]	XP_393061	11	25	104	6.83	51.44
21	major royal jelly protein 2 [Apis mellifera]	XP_393061	11	25	109	6.83	51.44
22	major royal jelly protein 2 [Apis mellifera]	XP_393061	11	25	113	6.83	51.44
23	major royal jelly protein 2 [Apis mellifera]	XP_393061	7	14	69	6.83	51.44
24	major royal jelly protein 2 [Apis mellifera]	XP_393061	8	18	59	6.83	51.44
25	major royal jelly protein 2 [Apis mellifera]	XP_393061	8	18	61	6.83	51.44
26	major royal jelly protein 2 [Apis mellifera]	XP_393061	4	10	43	6.83	51.44
27	major royal jelly protein 2 [Apis mellifera]	XP_393061	6	14	68	6.83	51.44
28	major royal jelly protein 2 [Apis mellifera]	XP_393061	5	12	51	6.83	51.44
29	major royal jelly protein 2 [Apis mellifera]	XP_393061	5	12	53	6.83	51.44
30	major royal jelly protein 2 [Apis mellifera]	XP_393061	5	12	48	6.83	51.44
31	major royal jelly protein 2 [Apis mellifera]	XP_393061	8	21	83	6.83	51.44
32	major royal jelly protein 2 [Apis mellifera]	XP_393061	8	21	76	6.83	51.44
33	major royal jelly protein 2 [Apis mellifera]	XP_393061	8	21	67	6.83	51.44
34	major royal jelly protein 3 [Apis mellifera]	XP_391893	6	18	53	6.47	61.62
35	major royal jelly protein 1 [Apis mellifera]	XP_393380	9	21	88	5.10	49.31
36	major royal jelly protein 1 [Apis mellifera]	XP_393380	8	18	74	5.10	49.31
37	major royal jelly protein 1 [Apis mellifera]	XP_393380	5	18	61	5.10	49.31
38	major royal jelly protein 1 [Apis mellifera]	XP_393380	5	18	54	5.10	49.31
39	major royal jelly protein 1 [Apis mellifera]	XP_393380	4	15	52	5.10	49.31
40	major royal jelly protein 1 [Apis mellifera]	XP_393380	4	15	54	5.10	49.31
41	major royal jelly protein 1 [Apis mellifera]	XP_393380	4	12	59	5.10	49.31
42	major royal jelly protein 1 [Apis mellifera]	XP_393380	7	20	84	5.10	49.31
43	major royal jelly protein 1 [Apis mellifera]	XP_393380	6	19	71	5.10	49.31
44	major royal jelly protein 1 [Apis mellifera]	XP_393380	6	19	68	5.10	49.31
45	major royal jelly protein 1 [Apis mellifera]	XP_393380	5	18	59	5.10	49.31
46	major royal jelly protein 1 [Apis mellifera]	XP_393380	5	18	58	5.10	49.31
47	major royal jelly protein 1 [Apis mellifera]	XP_393380	6	19	91	5.10	49.31
48	major royal jelly protein 1 [Apis mellifera]	XP_393380	6	19	65	5.10	49.31
49	major royal jelly protein 1 [Apis mellifera]	XP_393380	5	16	55	5.10	49.31
50	major royal jelly protein 2 [Apis mellifera]	XP_393061	4	11	54	6.83	51.44
51	major royal jelly protein 2 [Apis mellifera]	XP_393061	5	13	62	6.83	51.44
52	major royal jelly protein 2 [Apis mellifera]	XP_393061	6	15	79	6.83	51.44
53	major royal jelly protein 2 [Apis mellifera]	XP_393061	6	15	78	6.83	51.44
54	major royal jelly protein 2 [Apis mellifera]	XP_393061	6	15	71	6.83	51.44
55	major royal jelly protein 2 [Apis mellifera]	XP_393061	5	13	57	6.83	51.44
56	major royal jelly protein 2 [Apis mellifera]	XP_393061	5	13	55	6.83	51.44
57	major royal jelly protein 2 [Apis mellifera]	XP_393061	6	15	82	6.83	51.44
58	major royal jelly protein 2 [Apis mellifera]	XP_393061	4	11	56	6.83	51.44
59	major royal jelly protein 2 [Apis mellifera]	XP_393061	5	13	49	6.83	51.44
60	major royal jelly protein 2 [Apis mellifera]	XP_393061	6	15	74	6.83	51.44
61	major royal jelly protein 2 [Apis mellifera]	XP_393061	5	13	58	6.83	51.44
62	major royal jelly protein 3 [Apis mellifera]	XP_391893	6	19	98	6.47	61.62
63	major royal jelly protein 3 [Apis mellifera]	XP_391893	5	17	63	6.47	61.62
64	major royal jelly protein 3 [Apis mellifera]	XP_391893	7	21	88	6.47	61.62
65	major royal jelly protein 3 [Apis mellifera]	XP_391893	4	15	51	6.47	61.62
66	major royal jelly protein 3 [Apis mellifera]	XP_391893	5	17	60	6.47	61.62
67	major royal jelly protein 3 [Apis mellifera]	XP_391893	5	17	60	6.47	61.62
68	major royal jelly protein 3 [Apis mellifera]	XP_391893	6	19	71	6.47	61.62
69	major royal jelly protein 3 [Apis mellifera]	XP_391893	7	21	79	6.47	61.62
70	major royal jelly protein 3 [Apis mellifera]	XP_393380	7	21	86	6.47	61.62
71	profilin [Apis mellifera]	Q6QEJ7	3	47	77	5.65	13.72

a Protein spots excised from 2-DE (Figs. 6–7, gels A) were digested with trypsin and identified by MALDI-ToF MS analysis.

b Proteins were identified by MASCOT using the probability based MOWSE score (scores >53 are significant at *p*<0.05).

**Table 3:** Protein identification in the honey eucalyptus_MT1_ extract^a^.

Spot	Protein Name	Accession	Peptides	Cov. (%)	Score^b^	pI	kDa
1	major royal jelly protein 1 [Apis mellifera]	XP_393380	9	21	84	5.10	49.31
2	major royal jelly protein 1 [Apis mellifera]	XP_393380	7	16	59	5.10	49.31
3	alpha-glucosidase precursor [Apis mellifera]	XP_392790	6	16	71	5.06	65.69
4	Glucose oxidase [Apis mellifera]	XP_392386	10	20	98	6.48	67.90
5	Glucose oxidase [Apis mellifera]	XP_392386	8	19	63	6.48	67.90
5	major royal jelly protein 5[Apis mellifera]	XP_396121	7	11	51	5.90	70.19
6	major royal jelly protein 3 [Apis mellifera]	XP_393061	8	22	82	6.47	61.62
7	major royal jelly protein 2 [Apis mellifera]	XP_391893	11	27	139	6.83	51.44
8	major royal jelly protein 4 [Apis mellifera]	XP_001119880	6	14	66	5.89	52.88
9	major royal jelly protein 2 [Apis mellifera]	XP_393061	9	21	59	6.83	51.44
10	major royal jelly protein 2 [Apis mellifera]	XP_393061	9	21	71	6.83	51.44
11	major royal jelly protein 2 [Apis mellifera]	XP_393061	5	11	46	6.83	51.44
12	major royal jelly protein 3 [Apis mellifera]	XP_391893	13	32	153	6.47	61.62
13	major royal jelly protein 3 [Apis mellifera]	XP_391893	11	27	111	6.47	61.62
14	major royal jelly protein 3 [Apis mellifera]	XP_391893	8	22	75	6.47	61.62
15	major royal jelly protein 2 [Apis mellifera]	XP_393061	7	14	59	6.83	51.44
16	major royal jelly protein 2 [Apis mellifera]	XP_393061	12	33	172	6.83	51.44
17	major royal jelly protein 2 [Apis mellifera]	XP_393061	8	19	91	6.83	51.44
18	major royal jelly protein 2 [Apis mellifera]	XP_393061	9	21	105	6.83	51.44
19	major royal jelly protein 2 [Apis mellifera]	XP_393061	6	13	72	6.83	51.44
20	major royal jelly protein 3 [Apis mellifera]	XP_393061	10	25	109	6.47	61.62
21	major royal jelly protein 3 [Apis mellifera]	XP_393061	9	24	88	6.47	61.62
22	major royal jelly protein 3 [Apis mellifera]	XP_393061	7	16	64	6.47	61.62
23	major royal jelly protein 1 [Apis mellifera]	XP_393380	6	12	52	5.10	49.31
24	major royal jelly protein 1 [Apis mellifera]	XP_393380	6	12	61	5.10	49.31
25	major royal jelly protein 1 [Apis mellifera]	XP_393380	7	14	63	5.10	49.31
26	major royal jelly protein 2 [Apis mellifera]	XP_393061	4	10	50	6.83	51.44
27	major royal jelly protein 2 [Apis mellifera]	XP_393061	5	12	59	6.83	51.44
28	major royal jelly protein 2 [Apis mellifera]	XP_393061	6	14	58	6.83	51.44
29	major royal jelly protein 2 [Apis mellifera]	XP_393061	5	12	55	6.83	51.44
30	major royal jelly protein 2 [Apis mellifera]	XP_393061	5	12	57	6.83	51.44
31	major royal jelly protein 1 [Apis mellifera]	XP_393380	5	11	51	5.10	49.31
32	major royal jelly protein 1 [Apis mellifera]	XP_393380	7	14	81	5.10	49.31
33	major royal jelly protein 1 [Apis mellifera]	XP_393380	5	18	62	5.10	49.31
34	major royal jelly protein 1 [Apis mellifera]	XP_393380	5	18	61	5.10	49.31
35	major royal jelly protein 1 [Apis mellifera]	XP_393380	4	15	49	5.10	49.31
36	major royal jelly protein 1 [Apis mellifera]	XP_393380	4	15	51	5.10	49.31
37	major royal jelly protein 1 [Apis mellifera]	XP_393380	5	18	71	5.10	49.31
38	major royal jelly protein 1 [Apis mellifera]	XP_393380	4	12	43	5.10	49.31
39	major royal jelly protein 1 [Apis mellifera]	XP_393380	4	15	56	5.10	49.31
40	major royal jelly protein 1 [Apis mellifera]	XP_393380	4	12	52	5.10	49.31
41	major royal jelly protein 1 [Apis mellifera]	XP_393380	4	15	49	5.10	49.31
42	major royal jelly protein 2 [Apis mellifera]	XP_393061	5	13	61	6.83	51.44
43	major royal jelly protein 2 [Apis mellifera]	XP_393061	5	13	59	6.83	51.44
44	major royal jelly protein 2 [Apis mellifera]	XP_393061	5	13	57	6.83	51.44
45	major royal jelly protein 2 [Apis mellifera]	XP_393061	4	11	52	6.83	51.44
46	major royal jelly protein 2 [Apis mellifera]	XP_393061	4	11	50	6.83	51.44
47	major royal jelly protein 2 [Apis mellifera]	XP_393061	5	13	52	6.83	51.44
48	major royal jelly protein 2 [Apis mellifera]	XP_393061	5	13	55	6.83	51.44
49	major royal jelly protein 3 [Apis mellifera]	XP_393061	6	19	103	6.47	61.62
50	major royal jelly protein 3 [Apis mellifera]	XP_393061	6	19	82	6.47	61.62
51	major royal jelly protein 3 [Apis mellifera]	XP_393061	7	21	91	6.47	61.62
52	major royal jelly protein 3 [Apis mellifera]	XP_393061	5	17	77	6.47	61.62
53	major royal jelly protein 3 [Apis mellifera]	XP_393061	6	19	84	6.47	61.62
54	major royal jelly protein 3 [Apis mellifera]	XP_393061	5	17	61	6.47	61.62
55	Short chain dehydr/reduct [Apis mellifera]	F5HRF9	4	44	84	7.92	18.35

a Protein spots excised from 2-DE (Figs. 6–7, gels B) were digested with trypsin and identified by MALDI-ToF MS analysis.

b Proteins were identified by MASCOT using the probability based MOWSE score (scores >53 are significant at *p*<0.05).

**Table 4:** Protein identification in the honey chestnut_MT1_ extract^a^.

Spot	Protein Name	Accession	Peptides	Cov. (%)	Score^b^	pI	kDa
1	major royal jelly protein 1 [Apis mellifera]	XP_393380	14	32	172	5.10	49.31
2	alpha-glucosidase precursor [Apis mellifera]	XP_392790	7	19	77	5.06	65.69
3	n.d.						
4	n.d.						
5	major royal jelly protein 4 [Apis mellifera]	XP_001119880	8	18	69	5.89	52.88
6	major royal jelly protein 3 [Apis mellifera]	XP_393061	10	25	100	6.47	61.62
7	major royal jelly protein 3 [Apis mellifera]	XP_393061	7	19	84	6.47	61.62
8	major royal jelly protein 2 [Apis mellifera]	XP_391893	8	19	91	6.83	51.44
9	major royal jelly protein 1 [Apis mellifera]	XP_393380	6	13	47	5.10	49.31
10	major royal jelly protein 2 [Apis mellifera]	XP_393061	9	21	88	6.83	51.44
11	major royal jelly protein 2 [Apis mellifera]	XP_393061	9	21	75	6.83	51.44
12	major royal jelly protein 2 [Apis mellifera]	XP_393061	7	14	59	6.83	51.44
13	major royal jelly protein 2 [Apis mellifera]	XP_393061	7	14	57	6.83	51.44
14	major royal jelly protein 2 [Apis mellifera]	XP_393061	9	21	67	6.83	51.44
15	major royal jelly protein 2 [Apis mellifera]	XP_393061	7	16	53	6.83	51.44
16	major royal jelly protein 2 [Apis mellifera]	XP_393061	8	19	94	6.83	51.44
17	major royal jelly protein 2 [Apis mellifera]	XP_393061	8	19	117	6.83	51.44
18	major royal jelly protein 2 [Apis mellifera]	XP_393061	8	19	74	6.83	51.44
19	major royal jelly protein 2 [Apis mellifera]	XP_393061	6	14	59	6.83	51.44
20	major royal jelly protein 2 [Apis mellifera]	XP_393061	6	14	60	6.83	51.44
21	major royal jelly protein 3 [Apis mellifera]	XP_393061	4	10	42	6.47	61.62
22	major royal jelly protein 3 [Apis mellifera]	XP_393061	6	13	57	6.47	61.62
23	major royal jelly protein 1 [Apis mellifera]	XP_393380	5	18	69	5.10	49.31
24	major royal jelly protein 1 [Apis mellifera]	XP_393380	5	18	54	5.10	49.31
25	major royal jelly protein 1 [Apis mellifera]	XP_393380	5	18	51	5.10	49.31
26	major royal jelly protein 1 [Apis mellifera]	XP_393380	4	15	53	5.10	49.31
27	major royal jelly protein 1 [Apis mellifera]	XP_393380	4	15	51	5.10	49.31
28	major royal jelly protein 1 [Apis mellifera]	XP_393380	4	14	47	5.10	49.31
29	major royal jelly protein 1 [Apis mellifera]	XP_393380	4	12	44	5.10	49.31
30	major royal jelly protein 1 [Apis mellifera]	XP_393380	5	18	59	5.10	49.31
31	major royal jelly protein 1 [Apis mellifera]	XP_393380	4	12	50	5.10	49.31
32	major royal jelly protein 1 [Apis mellifera]	XP_393380	4	12	48	5.10	49.31
33	major royal jelly protein 1 [Apis mellifera]	XP_393380	4	14	46	5.10	49.31
34	major royal jelly protein 2 [Apis mellifera]	XP_393061	4	11	49	6.83	51.44
35	major royal jelly protein 2 [Apis mellifera]	XP_393061	5	13	62	6.83	51.44
36	major royal jelly protein 2 [Apis mellifera]	XP_393061	4	11	45	6.83	51.44
37	major royal jelly protein 2 [Apis mellifera]	XP_393061	6	15	66	6.83	51.44
38	major royal jelly protein 2 [Apis mellifera]	XP_393061	4	11	47	6.83	51.44
39	major royal jelly protein 2 [Apis mellifera]	XP_393061	4	11	44	6.83	51.44
40	major royal jelly protein 2 [Apis mellifera]	XP_393061	4	11	51	6.83	51.44
41	major royal jelly protein 3 [Apis mellifera]	XP_393061	7	19	98	6.47	61.62
42	major royal jelly protein 3 [Apis mellifera]	XP_393061	7	19	88	6.47	61.62
43	major royal jelly protein 3 [Apis mellifera]	XP_393061	7	19	76	6.47	61.62
44	major royal jelly protein 3 [Apis mellifera]	XP_393061	7	19	91	6.47	61.62
45	major royal jelly protein 3 [Apis mellifera]	XP_393061	7	19	64	6.47	61.62
46	major royal jelly protein 3 [Apis mellifera]	XP_393061	7	19	68	6.47	61.62
47	major royal jelly protein 3 [Apis mellifera]	XP_393061	6	17	65	6.47	61.62
48	major royal jelly protein 3 [Apis mellifera]	XP_393061	6	16	62	6.47	61.62
49	major royal jelly protein 3 [Apis mellifera]	XP_393061	6	13	57	6.47	61.62
50	major royal jelly protein 3 [Apis mellifera]	XP_393061	5	15	53	6.47	61.62
51	major royal jelly protein 3 [Apis mellifera]	XP_393061	6	17	59	6.47	61.62
52	major royal jelly protein 3 [Apis mellifera]	XP_393061	5	15	55	6.47	61.62
53	major royal jelly protein 3 [Apis mellifera]	XP_393061	5	14	60	6.47	61.62
54	Superoxide dismutase [Apis mellifera]	Q7YM6	5	58	107	6.21	15.63
55^c^	apimisin [Apis mellifera]	XP_393061	2	65	73	4.56	5.54

a Protein spots excised from 2-DE (Figs. 6–7, gels C) were digested with trypsin and identified by MALDI-ToF MS analysis.

b Proteins were identified by MASCOT using the probability based MOWSE score (scores >53 are significant at *p*<0.05).

c Protein spot was digested with chymotrypsin and identified by MALDI-ToF MS analysis.

**Table 5:** Protein identification in the honey sulla_MT1_ extract^a^.

Spot	Protein Name	Accession	Peptides	Cov. (%)	Score^b^	pI	kDa
1	major royal jelly protein 1 [Apis mellifera]	XP_393380	15	35	216	5.10	49.31
2	major royal jelly protein 1 [Apis mellifera]	XP_393380	11	25	139	5.10	49.31
3	alpha-glucosidase precursor [Apis mellifera]	XP_392790	8	21	92	5.06	65.69
4	Glucose oxidase [Apis mellifera]	XP_392386	7	17	54	6.48	67.90
5	Glucose oxidase [Apis mellifera]	XP_392386	7	17	59	6.48	67.90
6	major royal jelly protein 5 [Apis mellifera]	XP_396121	9	14	77	5.90	70.19
7	major royal jelly protein 3 [Apis mellifera]	XP_391893	13	30	114	6.47	61.62
8	major royal jelly protein 3 [Apis mellifera]	XP_391893	12	28	96	6.47	61.62
9	major royal jelly protein 4 [Apis mellifera]	XP_001119880	8	17	73	5.89	52.88
10	major royal jelly protein 2 [Apis mellifera]	XP_393061	10	22	85	6.83	51.44
11	major royal jelly protein 2 [Apis mellifera]	XP_393061	10	22	79	6.83	51.44
12	major royal jelly protein 2 [Apis mellifera]	XP_393061	9	21	81	6.83	51.44
13	major royal jelly protein 2 [Apis mellifera]	XP_393061	9	21	64	6.83	51.44
14	major royal jelly protein 2 [Apis mellifera]	XP_393061	6	14	55	6.83	51.44
15	major royal jelly protein 2 [Apis mellifera]	XP_393061	5	14	57	6.83	51.44
16	major royal jelly protein 2 [Apis mellifera]	XP_393061	12	33	108	6.83	51.44
17	major royal jelly protein 2 [Apis mellifera]	XP_393061	8	19	82	6.83	51.44
18	major royal jelly protein 2 [Apis mellifera]	XP_393061	9	21	84	6.83	51.44
19	major royal jelly protein 2 [Apis mellifera]	XP_393061	6	14	49	6.83	51.44
20	major royal jelly protein 2 [Apis mellifera]	XP_393061	7	15	59	6.83	51.44
21	major royal jelly protein 2 [Apis mellifera]	XP_393061	7	15	61	6.83	51.44
22	major royal jelly protein 2 [Apis mellifera]	XP_393061	7	15	53	6.83	51.44
23	major royal jelly protein 1 [Apis mellifera]	XP_393380	6	12	51	5.10	49.31
24	major royal jelly protein 2 [Apis mellifera]	XP_393061	7	15	56	6.83	51.44
25	major royal jelly protein 2 [Apis mellifera]	XP_393061	7	15	52	6.83	51.44
26	major royal jelly protein 2 [Apis mellifera]	XP_393061	5	10	39	6.83	51.44
27	major royal jelly protein 2 [Apis mellifera]	XP_393061	6	14	67	6.83	51.44
28	major royal jelly protein 2 [Apis mellifera]	XP_393061	6	14	68	6.83	51.44
29	major royal jelly protein 2 [Apis mellifera]	XP_393061	6	14	75	6.83	51.44
30	major royal jelly protein 2 [Apis mellifera]	XP_393061	5	12	45	6.83	51.44
31	major royal jelly protein 2 [Apis mellifera]	XP_393061	6	14	54	6.83	51.44
32	major royal jelly protein 2 [Apis mellifera]	XP_393061	8	21	83	6.83	51.44
33	major royal jelly protein 2 [Apis mellifera]	XP_393061	4	10	41	6.83	51.44
34	major royal jelly protein 2 [Apis mellifera]	XP_393061	5	12	47	6.83	51.44
35	major royal jelly protein 2 [Apis mellifera]	XP_393061	7	15	62	6.83	51.44
36	major royal jelly protein 1 [Apis mellifera]	XP_393380	5	18	66	5.10	49.31
37	major royal jelly protein 1 [Apis mellifera]	XP_393380	5	18	68	5.10	49.31
38	major royal jelly protein 1 [Apis mellifera]	XP_393380	5	18	59	5.10	49.31
39	major royal jelly protein 1 [Apis mellifera]	XP_393380	5	18	56	5.10	49.31
40	major royal jelly protein 1 [Apis mellifera]	XP_393380	4	14	46	5.10	49.31
41	major royal jelly protein 1 [Apis mellifera]	XP_393380	5	16	52	5.10	49.31
42	major royal jelly protein 1 [Apis mellifera]	XP_393380	4	14	50	5.10	49.31
43	major royal jelly protein 1 [Apis mellifera]	XP_393380	5	16	57	5.10	49.31
44	major royal jelly protein 1 [Apis mellifera]	XP_393380	4	14	52	5.10	49.31
45	major royal jelly protein 1 [Apis mellifera]	XP_393380	4	12	49	5.10	49.31
46	major royal jelly protein 1 [Apis mellifera]	XP_393380	4	12	44	5.10	49.31
47	major royal jelly protein 2 [Apis mellifera]	XP_393061	6	15	81	6.83	51.44
48	major royal jelly protein 2 [Apis mellifera]	XP_393061	6	15	76	6.83	51.44
49	major royal jelly protein 2 [Apis mellifera]	XP_393061	6	15	63	6.83	51.44
50	major royal jelly protein 2 [Apis mellifera]	XP_393061	6	15	61	6.83	51.44
51	major royal jelly protein 2 [Apis mellifera]	XP_393061	5	12	58	6.83	51.44
52	major royal jelly protein 2 [Apis mellifera]	XP_393061	5	13	62	6.83	51.44
53	major royal jelly protein 2 [Apis mellifera]	XP_393061	4	11	51	6.83	51.44
54	major royal jelly protein 2 [Apis mellifera]	XP_393061	4	11	43	6.83	51.44
55	major royal jelly protein 2 [Apis mellifera]	XP_393061	4	11	41	6.83	51.44
56	major royal jelly protein 3 [Apis mellifera]	XP_393061	7	19	88	6.47	61.62
57	major royal jelly protein 3 [Apis mellifera]	XP_393061	7	21	87	6.47	61.62
58	major royal jelly protein 3 [Apis mellifera]	XP_393061	7	21	66	6.47	61.62
59	major royal jelly protein 3 [Apis mellifera]	XP_393061	7	19	73	6.47	61.62
60	major royal jelly protein 3 [Apis mellifera]	XP_393061	5	16	66	6.47	61.62
61	major royal jelly protein 3 [Apis mellifera]	XP_393061	5	15	60	6.47	61.62
62	Superoxide dismutase [Apis mellifera]	Q7YM6	4	51	83	6.21	15.63

a Protein spots excised from 2-DE (Figs. 6–7, gels D) were digested with trypsin and identified by MALDI-ToF MS analysis.

b Proteins were identified by MASCOT using the probability based MOWSE score (scores >53 are significant at *p*<0.05).

Since the proteins of the MRJPs family constitute more than 90% of all honey proteins and this makes it difficult to detect and identify low-Mw minor proteins, electrophoretic runs were carried out on 15% polyacrylamide 2-D gels and then silver stained. Gels were intentionally over-stained in order to visualize the less abundant proteins. Several well-resolved spots were revealed in the low-Mw region of the gels.

Figure 7 shows the representative peptide profiles of the honey_MT1_ extracts corresponding to the ranges 4.0–9.0 for pI and molecular mass 14–30 kDa. The intrinsic proteolytic action against honey proteins is very clear and several spots were identified by MALDI-ToF mass spectrometry as fragments of some of the nine honey’s Major Royal Jelly Proteins (MRJPs).

**Figure 7:**
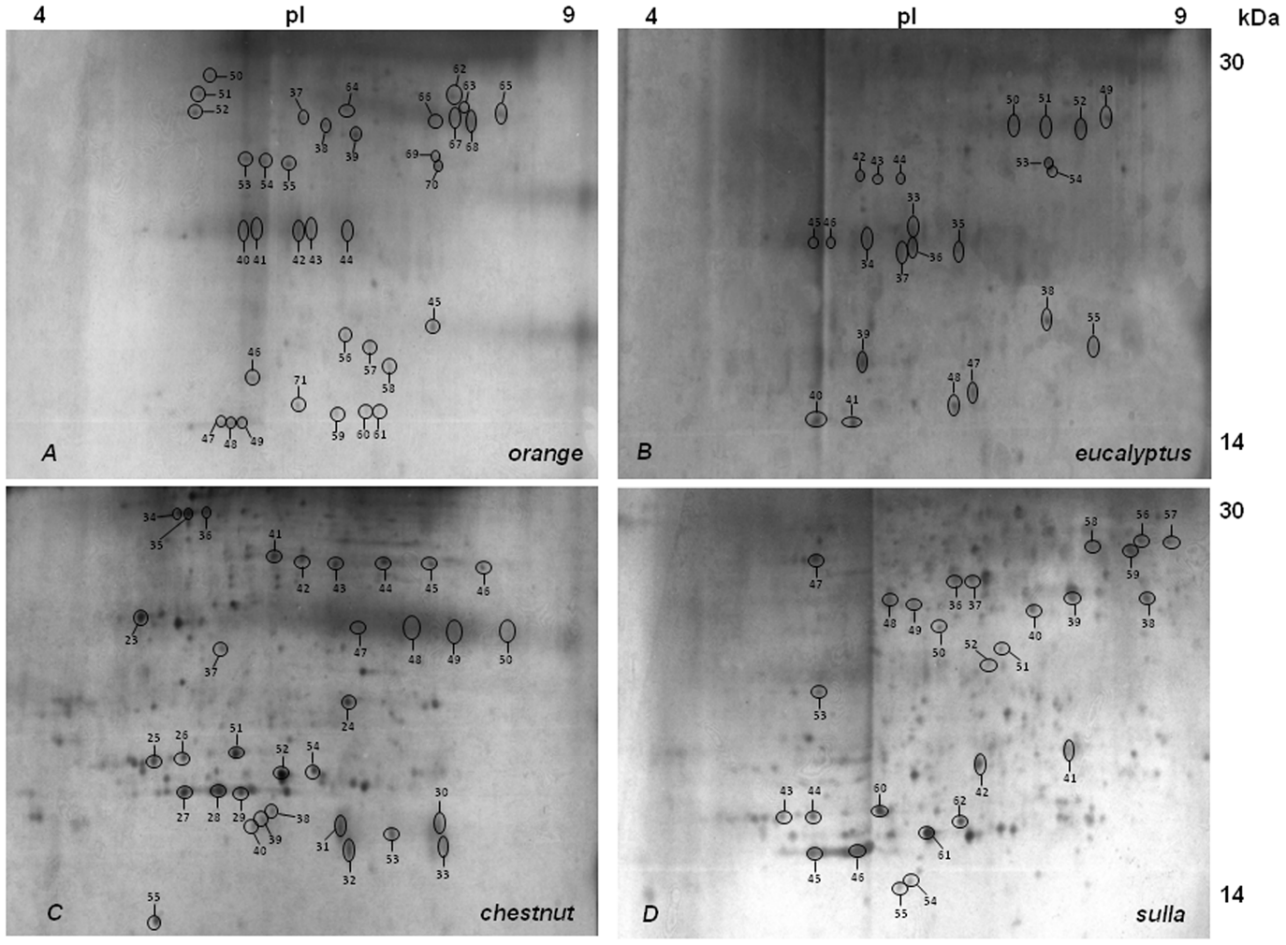
Bidimensional electrophoresis (2-DE) of proteins present in different unifloral honey_MT1_ samples. 2-DE analysis (IEF: linear pH gradient of 3–10; SDS-PAGE: 15% polyacrylamide). Silver stained gels: A (*orange*), B (*eucalyptus*), C (*chestnut*) and D (*sulla*). Only the regions of the gels with isoelectric point between pH 4–9 and apparent molecular mass between 14–30 kDa are shown. 60 µg of proteins were applied to each gel. Numbers corresponds to the protein spots identified by MALDI-ToF MS analysis.

As clearly shown in Fig. 7, in all honey samples, several peptides corresponding to MRJP1 (royalactin), MRJP2 and MRJP3 were detected. In particular, in *orange* (gel A), 13 spots were identified as MRJP1 fragments (spots 37–49), 12 spots were identified as MRJP2 fragments (spots 50–61) and 9 as MRJP3 fragments (spots 62–70) (Table 2). Whereas, in *eucalyptus* (gel B), 9 spots were identified as MRJP1 fragments (spots 33–41), 7 spots were identified as MRJP2 fragments (spots 42–48) and 6 as MRJP3 fragments (spots 49–54) (Table 3). In *chestnut* (gel C), 11 spots were identified as MRJP1 fragments (spots 23–33), 7 spots were identified as MRJP2 fragments (spots 34–40) and 13 as MRJP3 fragments (spots 41–53) (Table 4). Finally, in *sulla* (gel D), 11 spots were identified as MRJP1 fragments (spots 36–46), 9spots were identified as MRJP2 fragments (spots 47–55) and 6 as MRJP3 fragments (spots 56–61) (Table 5).

In addition to the proteolytic fragments deriving from MRJPs, other 4 proteins belonging to the proteome of *Apis mellifera* were identified. In particular, Profilin (AC Q6QEJ7, Mw 13.72, pI 5.64) in *orange* (spot 71); Short-Chain Dehydrogenase/Reductase (AC F5HRF9, Mw 18.35, pI 7.92) in *eucalyptus* (spot 55); Superoxide Dismutase (AC Q7YXM6, Mw 15.63, pI 6.21) in *chestnut* (spot 54) and in *sulla* (spot 62); Apimisin (AC Q8ISL8, Mw 5.54, pI 4.56) in *chestnut* (spot 55), this latter was identified after digestion of the spot with chymotrypsin.

Spots corresponding to the proteolytic enzymes were not identified, probably due to their very low amounts, which cannot be detected, whereas their activity can be detect by zymography, a very sensitive technique that allows the detection of the activity of enzymes in the order of ng. Proteins of plant origin were not detected, probably because they were degraded by honeybee proteases and were present in undetectable amounts.

Our data indicate that all the proteins that can be detected by 2-D gel electrophoresis belongs to the *Apis mellifera* proteome. Our results are consistent with those reported in a recent work, showing that all proteins, except one, from five different honeys (*acacia, eucalyptus, orange, chestnut* and *sunflower*) belonged to the *Apis mellifera* proteome [26].

Our results suggest that the proteolytic enzymes of honey can significantly change honey protein profile and thereby strongly influence quality and nutritional value of honey and royal jelly. This latter is the food of the queen honeybee larva and contains special factors driving its development. The most important among these factors is MRPJ1. This protein (also called royalactin) has been recently reported to be the factor responsible of queen development through an Egfr-mediated signalling pathway [27]. Its degradation might be relevant for its activity. In this light, the detection of honey proteolytic enzymes might open new perspectives in different fields of research and in the handling of honey and royal jelly.

In conclusion, we report here on the usage of bidimensional zymography to detect for the first time all the honey proteolytic enzymes active against gelatin.

The observed zymographic patterns were typical for each floral variety, but they were found to correspond to the honeybees at work and not to the floral origin. Since floral proteins were not detectable also in the corresponding 2-DE runs, results suggest that floral substrates are not directly relevant for honey composition, but they can influence the enzymatic pattern of honeys and thereby the metabolism of honeybees in general. Specific induction of the expression of proteolytic enzymes of honeybees is strongly related to the degradation of honey proteins, and the consequent change of the activity of royal jelly factors could influence honeybee development and social behaviour. Therefore, evaluation of the proteolytic network of honeys by 2-DZ may be useful in the assignment of floral origin of honeys, since they are much easier to evaluate than 2-DE for the characterization of honeys, and may allow a better understanding of the mechanisms involved in honey manufacturing.

## Materials and Methods

### Chemicals

All reagents used were of the highest grade and were purchased from Sigma-Aldrich (St. Louis, MO, USA), Carlo Erba (Milan, Italy), Bio-Rad Laboratories (Segrate, Italy) and GE Healthcare (Uppsala, Sweden).

### Honey Samples

In this study were analyzed 16 commercial unifloral honey samples from 4 different floral origin: orange (*Citrus* sp), chestnut (*Castanea sati*v*a* Miller.), eucalyptus (*Eucalyptus* sp) and Italian sainfoin (*Hedysarium coronarium* L.) commonly known in Italy as “sulla”. Their botanical origin was ascertained by palynological analysis [28]. Honeys were manufactured by native Italian honeybees (*Apis mellifera ligustica*) in fields of the two different geographical origins in the Provinces of Matera and Potenza (Basilicata, Southern Italy). For each honey type, four samples were analyzed, two manufactured in the Province of Matera (honey_MT1_ and honey_MT2_) and two in Province of Potenza (honey_PZ1_ and honey_PZ2_). Honey samples were stored at 4°C in the dark and analyzed six months after their production.

### Enzyme Extraction and Protein Assay

For enzyme extraction, two aliquots of 5 g of honey (stored at 20°C) were mixed with an equal volume of 50 mM Tris/HCl, pH 7.5, and suspended at 4°C for 4 h. After centrifugation at 10,000×g for 10 min at 4°C, the supernatants (10 mL) were dialyzed (membrane cutoff 3,500 Da, Spectrum Laboratories, Inc.) against 2 L of distilled water for 24 h at 4°C, dried in speed-vac and stored at −70°C. Protein concentration was determined according to the method of Bradford [29] and the Bio-Rad reagent, using bovine serum albumin as standard protein.

### Assessment of Proteolytic Activity in Solution

Proteolytic activity extracted from honey samples was determined in solution using gelatin as substrate. Briefly, aliquots of freeze-dried extracts (corresponding to 1 mL of dialyzed extracts) were resuspended in 100 µL of 40 mM Tris-HCl buffer pH 7.5 and added to 350 µL of 1% (w/v) gelatin in 40 mM Tris-HCl buffer, pH 7.5. After incubation for 60 min at 30°C the reaction was stopped by the addition of 450 µL of 4% TCA. The mixtures were centrifuged for 10 min at 13,000 rpm (Amicon microcentrifuge MC-13), and the absorbance of the supernatants was measured at 280 nm. The assay included an appropriate blank, represented by the enzyme solution to which TCA was added before the substrate.

Proteolytic activity was expressed as mU mg^−1^ of proteins, where one milliunit of total proteolytic activity (mU) was the amount of enzyme yielding 0.001 unit of absorbance per min. Each analysis was done in triplicate and data are presented as mean ± standard deviation (n = 6). One-way ANOVA was carried out to test for significant differences and results were considered to be statistically significant at *p*<0.05.

### 2-D Gelatin Zymography (2-DZ)

Samples were applied under non-reducing conditions. Aliquots of freeze-dried extracts (80 µg of proteins) were mixed with the rehydration solution containing 7 M urea, 2 M thiourea, 2% (w/v) CHAPS, 0.5% (v/v) IPG (Immobilized pH Gradient) buffer, plus a trace of bromophenol blue, to a final volume of 250 µL. IEF (Isoelectrophocusing) was performed on IPG Dry-Strips of 13 cm in linear pH gradient of 3–10 (GE-Healthcare). IPG Dry-Strips were rehydrated with a sample-containing rehydration solution for 10 h at 20°C. IEF was run using an IPGphor unit (Amersham Biosciences) at 20°C for a total of 32,450 Vh. After IEF, IPG-strips were equilibrated for 20 min by gentle shaking in equilibration buffer: 6 M urea, 30% (w/v) glycerol, 2% (w/v) SDS, 50 mM Tris-HCl (pH 8.8). In the second dimension proteins were separated in a 15% (w/v) polyacrylamide gel copolymerized with 0.1% (w/v) gelatin. Run was carried out on the Hoefer SE 600 vertical electrophoresis unit at 4°C first for 30 min at 200 V and then for 5 h at 280 V. Pre-stained standard protein (National Diagnostic) were used as molecular weight markers. For the inhibition studies, specific inhibitors were added to the freeze-dried extracts and incubated for 1 h at 20°C before electrophoresis. Final concentrations of the inhibitors were: 1.5 mM phenylmethylsulphonyl fluoride (PMSF) for serine proteinases; 30 mM iodoacetamide for cysteine proteinases; and 20 mM ethylenediamine-tetraacetic acid (EDTA) for metallo-proteinases.

After electrophoresis, gels were washed two times in 2.5% (w/v) Triton X-100, 40 mM Tris-HCl buffer (pH 6.8) and then incubated for 14 h at 30°C in developing buffer: 1% (w/v) Triton X-100, 2 mM DTT, 10 mM cysteine, 5 mM CaCl_2_, 40 mM Tris-HCl buffer (pH 6.8), in absence and in presence of specific inhibitors. For the development of enzymatic activities, gels were stained with Coomassie Brilliant Blue R-250 for 30 min at room temperature, and destained in methanol/acetic acid/H_2_O. Proteolytic activity was detected as a clear, unstained spot on a blue background.

Gels were scanned using a ScanMaker 9800 XL-Microtek (Hsinchu, Taiwan). The images were digitally converted from positive to negative image. Image analysis for the determination of apparent Mr and pI, was carried out using the ImageMaster 2D Elite V. 2002.01 software (Amersham Biosciences).

### 2-D Polyacrylamide Gel Electrophoresis (2-DE)

Honey samples were extracted in the presence of a cocktail of protease inhibitors (Sigma), dialyzed, precipitated with 10 volumes of ice-cold acetone containing 10% (w/v) trichloroacetic acid (TCA) and 20 mM DTT, and kept at −20°C overnight. After centrifugation (3,000 g, 10 min, 4°C), the pellets were washed twice with 1 mL of ice-cold acetone containing 20 mM DTT, dried and stored at −70°C.

Aliquots of freeze-dried extracts (60 or 300 µg of proteins) were mixed with 250 µL of rehydration solution containing 7 M urea, 2 M thiourea, 2% (w/v) CHAPS, 60 mM DTT, 0.5% (v/v) IPG buffer, plus a trace of bromophenolblue.IEF was carried out as described before for 2-DZ.After IEF, the IPG-strip equilibration step was carried out for 20 min in 1% (w/v) DTT containing equilibration buffer and then for 20 min in the same solution with 4% (w/v) iodoacetamide plus a trace of bromophenol blue. Proteins were separated in the second dimension (SDS-PAGE) in a 10% (w/v) polyacrylamide (for Coomassie staining) and 15% (w/v) polyacrylamide (for silver staining) gels, respectively. Runs were carried out as described for 2-DZ. Gels were stained according to the protocol described by Shevchenko et al. [30] (1996), which is compatible with mass spectrometry analysis. Gels were digitized as 600 dpi, 16 bit TIFF images and imported to the ImageMaster 2D Elite software.

### Protein Identification by MALDI-ToF Mass Spectrometry (MS)

Protein spots were excised from 2-DE gels, destained and digested with trypsin. The extracted tryptic fragments were mixed with the matrix solution: 1% (w/v) α-cyano-4-hydroxy-cinnamic acid solution, 50% (v/v) acetonitrile, 0.5% (v/v) trifluoroacetic acid, and analyzed by MALDI-ToF MS. Mass spectra were acquired in positive reflectron mode at 20 kV using an Ettan MALDI-ToF Pro mass spectrometer (Amersham Biosciences) equipped with an UV nitrogen laser (337 nm) with delayed extraction mode and low mass rejection. For each spectrum 256 single shots were accumulated. Peptide spectra were calibrated using two standard peptides (ile-7AngIII, M+H 897.531, monoisotopic, and hACTH 18–39, M+H 2465.191, monoisotopic). Protein identification was performed by the MASCOT search engine (http://www.matrixscience.com) against the NCBInr protein and Swiss-Prot/TrEMBL databases using peptide mass fingerprinting (PMF). The following parameters were used for database search: 1) taxonomy group: *Viridiplantae* (Green Plants) and Other Green Plants or *Metazoa*; 2) mass tolerance of 0.2 Da, 3) one missed tryptic cleavage allowed, 4) carboamidomethylation of cysteine (as a fixed modification) and 5) oxidation of methionine (as a variable modification). Proteins were identified by MASCOT using the probability-based MOWSE score, equal to -10XLog(P), where P is the probability that the observed match is a random event. Protein scores of >53 were considered statistically significant (P<0.05) under the selected variables.
